# Parametric Study of Flexural Strengthening of Concrete Beams with Prestressed Hybrid Reinforced Polymer

**DOI:** 10.3390/ma12223790

**Published:** 2019-11-18

**Authors:** Xiaomeng Wang, Michal Petrů, Jun Ai, Shikun Ou

**Affiliations:** 1 Institute for Nanomaterials, Advanced Technologies and Innovation, Technical University of Liberec, Studentska 2, 461 17 Liberec, Czech Republic; Xiaomeng.Wang@tul.cz; 2 College of Aerospace Engineering, Nanjing University of Aeronautics and Astronautics, Nanjing 210016, China; aijun0822@nuaa.edu.cn (J.A.); oushikun@yeah.net (S.O.)

**Keywords:** hybrid reinforced composite, strengthening, reinforced concrete beams, numerical investigation

## Abstract

The strengthening method of using hybrid fiber reinforced polymer is an effective way to increase the strengthening efficiency and lower the cost. This paper focuses on simulating the flexural behavior of reinforced concrete beam strengthened by prestressed C/GFRP (Carbon-Glass hybrid Fiber Reinforced Polymer) with different hybrid ratios and prestress levels. An elastoplastic damage constitution is used to simulate the mechanical behavior of concrete. A cohesive zone model under mixed mode is adopted to describe the debonding behavior of the FRP-concrete and concrete-steel interface. The results show good agreement with the experiment in the load-deflection curve, load-stress curve of steel, and HFRP. Furthermore, the failure mode of concrete and FRP debonding obtained from numerical simulation is the same as the test. Considering the improvement of the bending capacity, stiffness, and ductility of the strengthened beam in this paper, the best hybrid ratio of carbon to glass fiber is 1:1, and the suitable prestress level is between 30 and 50% of its ultimate strength.

## 1. Introduction

Environmental factors, material aging, and overloading may affect the bearing capacity of reinforced concrete structures (such as beams, columns, slabs, etc.). Compared with demolition and reconstruction, FRP (Fiber Reinforced Polymer) strengthening can reduce costs and optimize investment. CFRP (Carbon Fiber Reinforced Polymer) is the most commonly used FRP. The modulus and strength of CFRP are higher compared to the values of GFRP (Glass Fiber Reinforced Polymer), but the drawback is that the elongation of CFRP is relatively low, which makes the failure mode of the reinforced concrete structure brittle. Besides, the price of CFRP is very high. If high elongation and less expensive fiber such as glass fiber can be mixed with CFRP, not only the strength and elongation of HFRP (Hybrid Fiber Reinforced Polymer) can be improved, but the cost can be significantly reduced.

Experiments were performed to investigate the strengthening effect of HFRP. Attari et al. [1], Hawilleh et al. [2], and Xiong et al. [3] studied the performance of concrete beams strengthened by HFRP. The effectiveness, durability, and economy of the HFRP strengthening method were discussed. Attari et al. [1] reported that the capacity of the beam strengthened by C/GFRP (Carbon/Glass hybrid Fiber Reinforced Polymer) increases significantly, and the increase does not come with brutal ductility loss. Hawilleh et al. [2] found that the increase of the bending capacity of the strengthened beams ranged from 30% to 98% of the reference beam, depending on the mix ratio of carbon fiber and glass fiber in HFRP. Xiong et al. [3] compared the economy of CFRP and C/GFRP strengthening. Results showed that the cost of C/GFRP is 38% less than that of CFRP. Chen et al. [4] compared the flexural fatigue properties of RC (Reinforced Concrete) beams strengthened with carbon/glass hybrid FRP, carbon/basalt fiber hybrid FRP, and BFRP (Basalt Fiber Reinforced Polymer), and the results showed that the fatigue life of the beam strengthened with carbon/glass hybrid FRP was the longest. He et al. [5] studied the anti-cracking effect of carbon/glass hybrid FRP strengthened concrete beams and discussed the strengthening mechanism of hybrid fibers. It was noted that the C/GFRP had a good impact on the ductility of strengthened RC beams, and the strengthened RC beam exhibited higher cracking load and ultimate load capacity than the reference beam.

In the aspect of the numerical simulation of FRP strengthened concrete structures, most of the studies focused on the modeling and analysis of uncracked concrete structures strengthened by FRP [6], while numerical research on FRP strengthened pre-cracked structure [7] is limited. In the numerical simulation, Hu et al. [6] and Bennegadi et al. [8] adopted the method of joint consolidation to deal with the FRP/concrete interface. Hawileh [9] used spring elements to simulate the FRP/concrete interface. Xu et al. [10] and Obaidat [11] compared three methods of joint consolidation, the spring element, and cohesive element to simulate the interface between FRP and concrete. The results of the spring element and cohesive element were closer to the test results. Bennegadi et al. [8] carried out numerical simulation to optimize the height and width of the HFRP used in beam strengthening. The results showed that the most important optimization factor is the thickness of the FRP. It is necessary not to reduce the length significantly, in order to stabilize the stress concentration at the edges of the FRP plate and to avoid debonding near the interface.

In order to take full advantage of FRP, it is necessary to have a good understanding of the failure mechanism of each component material and the failure process of the strengthened structure. Therefore, in this paper, the constitutive models of concrete and interface are proposed and implemented by the user subroutine UMAT of the finite element software Abaqus, and the strengthening effect of HFRP is numerically studied.

## 2. Constituent Materials

### 2.1. Constitutive Response of Concrete

The mechanical behavior of concrete under uniaxial compressive stress is different from that under multiaxial compressive stress [12]. The strength of concrete is different when it is subjected to compression and tension. The tensile strength of the concrete is far less than its compressive strength. The material model of concrete is available in the commercial software Abaqus (6.10). However, there are some limitations of the model in Abaqus, such as the convergence problem of concrete constitutive response in the softening phase [13]. In addition, the damage factor of concrete in the model of Abaqus is obtained by fitting the stress-strain curve of the test, which cannot truly reflect the relationship between plastic strain and the damage factor. In this paper, an isotropic elastic-plastic damage model is used to simulate the non-linear mechanical behavior of concrete under multiaxial stress. The constitutive model of concrete, which combines plasticity with damage, can reflect not only the stiffness degradation caused by damage, but also the strain softening and plastic deformation [14]. Shen et al. [15] proposed a plastic damage model of concrete with reference to the generalized Drucker-Prager criterion, and the plastic damage loading condition is primarily defined in the effective stress space. The inelastic potential function of the isotropic elastic-plastic damage model of concrete *F* (Equation (1)) includes two parts: plastic potential ${\tilde{Q}}_{q}$ (Equation (2)) and damage potential *Q_d_* (Equation (3)) [15].

(1) $$F={\tilde{Q}}_{q}+Q_{d}$$

(2) $${\tilde{Q}}_{q}=\alpha {\tilde{I}}_{1}+\sqrt{{\tilde{J}}_{2}}$$

(3) $$Q_{d}=\frac{S\gamma }{2{\left(1-D\right)}^{\phi }}{\left(\frac{Y}{S\gamma }\right)}^{2}$$

(4) $$Y=(1-D)E\varepsilon_{}^{e}\varepsilon_{}^{e}$$

(5) $$\gamma =\left|{\tilde{I}}_{1}/\sqrt{2{\tilde{J}}_{2}/3}\right|$$

As concrete is a porous material with volume compressibility, the yield of concrete is controlled by both effective stress and hydrostatic stress. In Equation (2), α is the pressure sensitive property constant of concrete, ${\tilde{I}}_{1}$ is the first stress invariant of the effective stress tensor, and ${\tilde{J}}_{2}$ is the second stress invariant of the effective partial stress tensor. Y (Equation (4)) is the release rate of damage strain energy. *D* is damage variable. γ (Equation (5)) is the stress triaxial ratio. *S* is a material parameter describing the softening behavior of concrete, and *ϕ* is the damage evolution parameter.

In this model, damage evolution is coupled with plastic strain. Plasticity and damage evolution are described as follows [16].

(6) $$\Delta \varepsilon_{p}=\Delta \lambda \frac{\partial {\tilde{Q}}_{q}}{\partial \sigma }$$

(7) $$\Delta D=\Delta \lambda \frac{\partial Q_{d}}{\partial Y}$$

where Δ*λ* is a plastic damage operator, which can be calculated from the plastic damage loading condition as:

(8) $$\tilde{f}=\alpha {\tilde{I}}_{1}+\sqrt{{\tilde{J}}_{2}}-\sigma_{y}^{}=0$$

where σ_y_ is the yield function, and the Newton–Raphson iteration expression is:

(9) $${\tilde{f}}_{i}+\frac{\partial \tilde{f}}{\partial (\Delta \lambda )}(\Delta \lambda_{i+1}-\Delta \lambda_{i})=0$$

The stress increment can be obtained as:

(10) $$\Delta \sigma =\frac{\partial \sigma }{\partial D}\Delta D+\frac{\partial \sigma }{\partial \varepsilon }\Delta \varepsilon$$

Considering the plastic consistency condition, the relationship between Δλ and Δ**ε** can be obtained as $\Delta \lambda =\frac{A}{B}\Delta \varepsilon$. The elastic-plastic matrix of the material can be obtained by substituting it into Equation (10).

(11) $$E^{ep}=E{\left(1-D\right)}^{2}-\frac{A}{B}[E{\left(1-D\right)}^{2}n+2E(1-D)\varepsilon^{e}\bar{Y}]$$

(12) $$\bar{Y}=\frac{\partial Q_{d}}{\partial Y}=\frac{Y}{S\gamma {\left(1-D\right)}^{\phi }}$$

where $A=\frac{\partial {\tilde{Q}}_{q}}{\partial \sigma }E{\left(1-D\right)}^{2}$, $B=\frac{\partial {\tilde{Q}}_{q}}{\partial \sigma }:[E{\left(1-D\right)}^{2}:\frac{\partial {\tilde{Q}}_{q}}{\partial \sigma }+2E(1-D):\varepsilon^{e}]-\frac{\partial {\tilde{Q}}_{q}}{\partial \lambda }-\frac{\partial {\tilde{Q}}_{q}}{\partial D}\bar{Y}$, $\bar{Y}$ is the energy release rate density [15].

**Figure 1:**
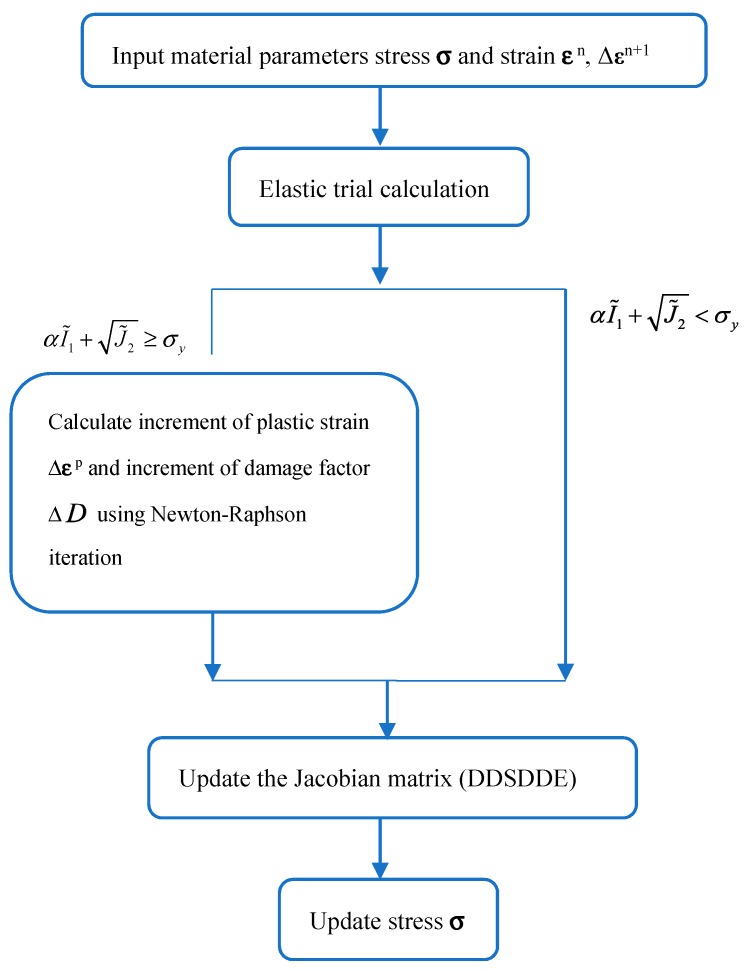
Flowchart of the plastic damage model of concrete.

**Figure 2:**
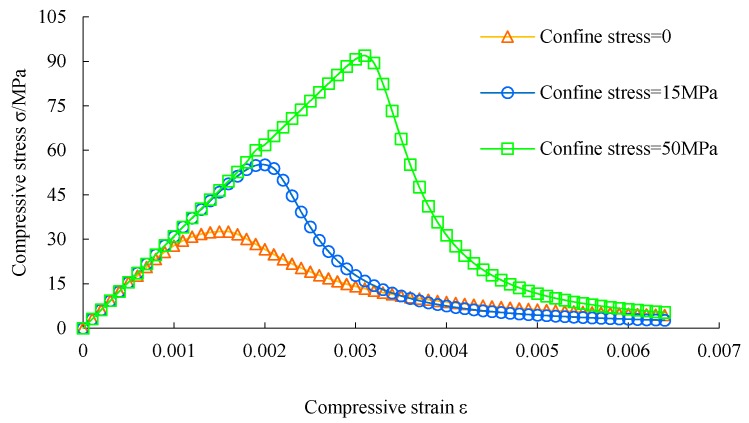
Stress-strain curve of concrete under compression.

**Figure 3:**
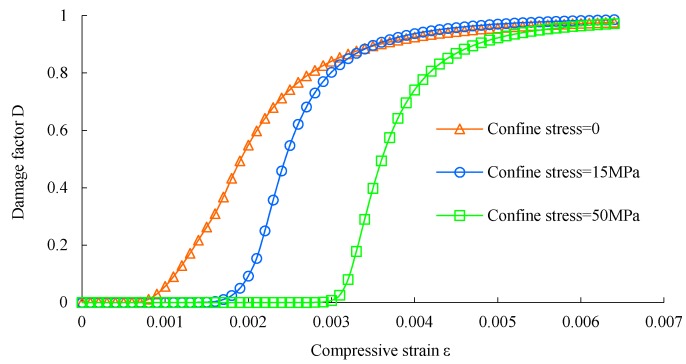
Damage factor of concrete under compression.

**Figure 4:**
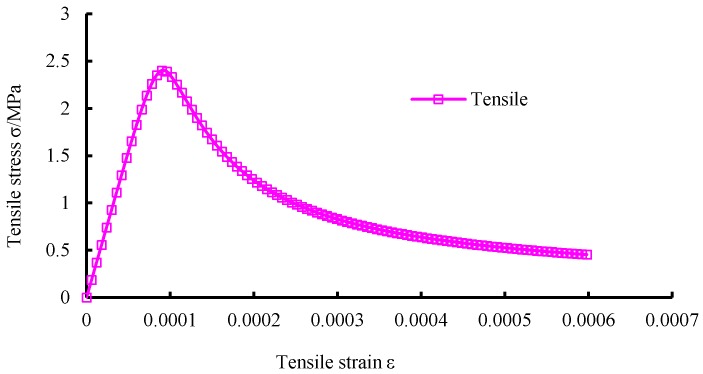
Stress-strain curve of concrete under tension.

The flowchart of the plastic damage model of concrete is shown in Figure 1. The mechanical properties of concrete are listed in Table 1. The mechanical behavior of concrete under compression and tension is shown in Figure 2, Figure 3 and Figure 4, respectively. The elastic modulus and strength were chosen according to the test results of [17]. The material parameter describing the damage of concrete was adopted according to [16]. 

### 2.2. Constitutive Response of the Interface in Mixed Mode

**Figure 5:**
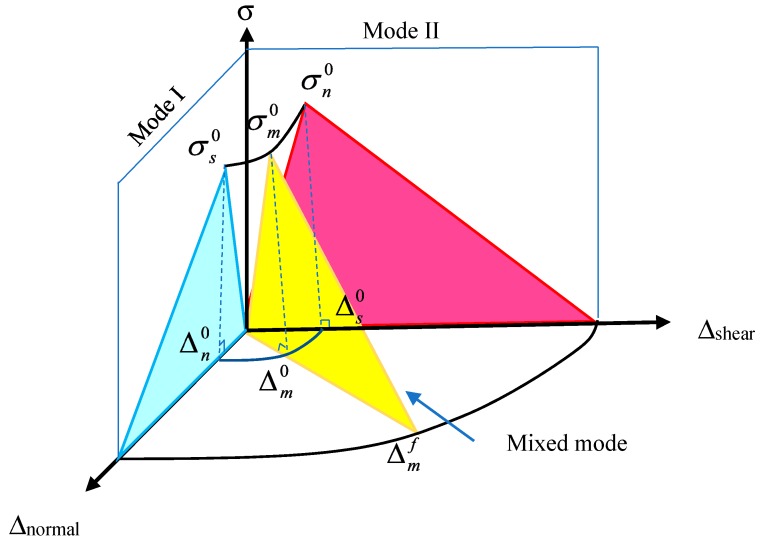
Constitution of interface for mixed-mode delamination.

The Cohesive Zone Model (CZM) [18] was used to simulate the concrete/FRP and concrete/steel interface. The CZM constitutive model consists of three displacement jumps (interfacial separation) Δ_i_, corresponding to three engineering stresses σ_i_, where Δ_1_ and Δ_2_ are displacement jumps in the shear direction, and the equivalent shear displacement jump is $\Delta_{s}=\sqrt{\Delta_{1}^{2}+\Delta_{2}^{2}}$. Δ_3_ is the displacement jump in the normal direction, $\Delta_{n}=\Delta_{3}$. The tangential and normal displacements are coupled, and the equivalent displacement jump $\Delta_{m}=\sqrt{\Delta_{s}^{2}+\Delta_{n}^{2}}$ is introduced to represent the total displacement, as shown in Figure 5.

When the interface is in a uniaxial compression state, the interface will not debond. Debonding of the interface is trigged by tensile stress or shear stress. In the actual engineering structure, the interface is always in the state of mixed loading in which tensile and shear stress exist at the same time. Therefore, it is necessary to determine the failure criteria of the interface in the mixed mode. The modulus of the cohesive zone model in Abaqus is the same in the normal and shear direction. However, the modulus of FRP/concrete and FRP/steel interface in the normal and shear direction is different. Therefore, the constitutive relation of the interface under mixed mode is:

(13) $$\sigma =\left\{\begin{array}{l} \sigma_{1}\\ \sigma_{2}\\ \sigma_{3} \end{array}\right\}=\left(1-d\right)\left\{\begin{array}{l} K_{s}\Delta_{1}\\ K_{s}\Delta_{2}\\ K_{n}\Delta_{3} \end{array}\right\}-d\left\{\begin{array}{l} 0\\ 0\\ K_{n}\langle \Delta_{3}\rangle \end{array}\right\}$$

where *K* is the penalty stiffness and $d=\frac{\Delta_{m}^{f}\left(\Delta_{m}-\Delta_{m}^{0}\right)}{\Delta_{m}\left(\Delta_{m}^{f}-\Delta_{m}^{0}\right)}$ is the damage factor. $\Delta_{m}^{0}$ is the initial damage threshold. $\Delta_{m}^{f}$ is the failure displacement jump corresponding to complete debonding. $\Delta_{m}^{0}$ is determined by damage criteria as follows [19].

(14) $${\left(\frac{\langle \Delta_{n}\rangle }{\Delta_{n}^{0}}\right)}^{2}+{\left(\frac{\Delta_{s}}{\Delta_{s}^{0}}\right)}^{2}=1$$

**Figure 6:**
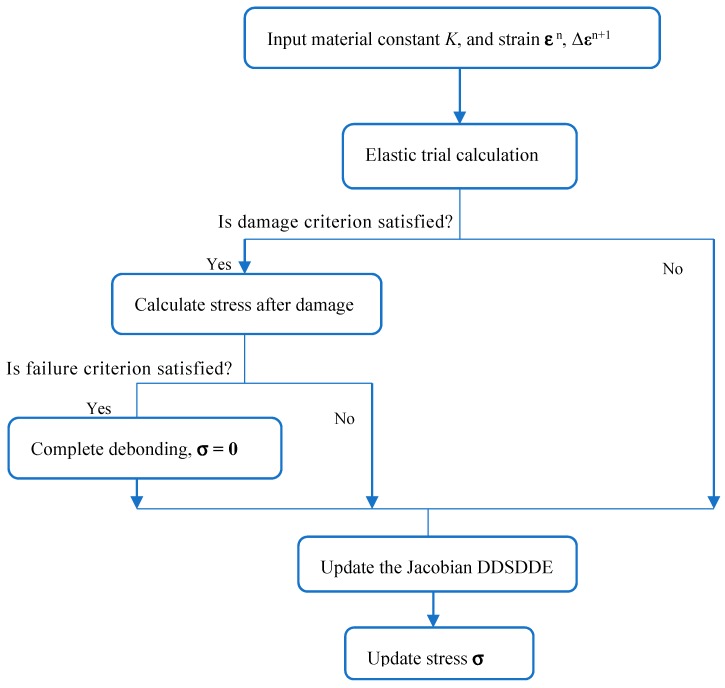
Flowchart for the constitutive model of the interface.

The $\Delta_{m}$ composed by them is defined as $\Delta_{m}^{0}$. Here, $\Delta_{n}^{0}$ and $\Delta_{s}^{0}$ are the damage initiation displacement jumps for Pure Mode I and Mode II. $\Delta_{m}^{f}$ is obtained by debonding criteria as follows [20].

(15) $$\Delta_{m}^{f}=\frac{2\left(1+\gamma^{2}\right)}{\Delta_{m}^{0}}{\left[{\left(\frac{K_{n}}{G_{n}^{0}}\right)}^{\eta }+{\left(\frac{\gamma^{2}K_{s}}{G_{s}^{0}}\right)}^{\eta }\right]}^{-1/\eta }$$

where $G_{n}^{0}$ and $G_{s}^{0}$ are the fracture energies of the interface corresponding to Mode I and Mode II, and they equal the areas of the triangles in the coordinate planes of $T-O-\Delta_{n}$ and $T-O-\Delta_{s}$, respectively. $\gamma =\frac{\Delta_{s}}{\Delta_{n}}$ is the displacement based mode-mixity ratio, and *η* is a material constant. The flowchart of the constitutive of interface in mixed mode is shown in Figure 6. The mechanical properties of the interface were obtained according to [21] and listed in Table 2 and Table 3. The material parameters of steel bar and epoxy used to determine the interfacial properties were the same as [21].

### 2.3. Constitutive Model of Steel and HFRP

**Figure 7:**
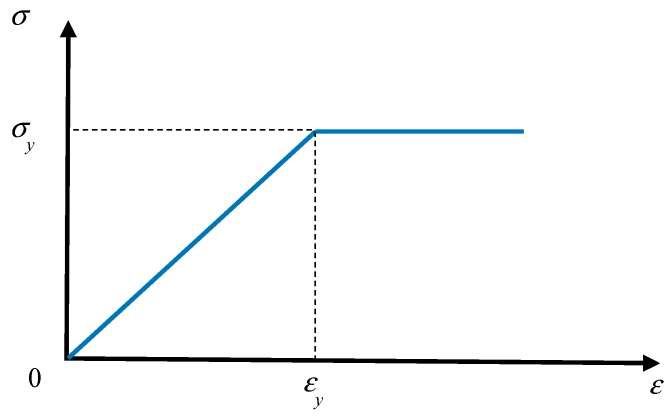
Stress-strain curve of steel.

The elastic-plastic model of the steel bar is shown in Figure 7 [22], where σ_y_ is its yield strength and ε_y_ is the yield strain. In the finite element model of this paper, the strain of the steel bar does not exceed the hardening strain. The yield criterion for steel is the Mises criterion. The mechanical properties of steel were obtained from the tensile test of a steel bar sample with a length of 500 mm of Ou [17] and listed in Table 4.

The mechanical behavior of HFRP is supposed to be elastic, and the mechanical properties are shown in Table 5 [23]. The Poisson ratio of HFRP was 0.3 in the longitudinal direction of the fiber and 0.17 in the other direction [24].

## 3. Finite Element Model of RC Beam Strengthened by Prestressed HFRP

**Figure 8:**
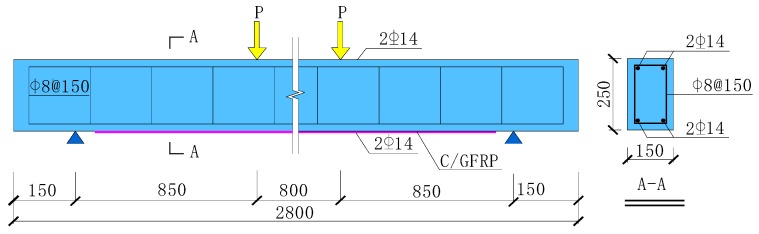
Configuration of the test beam (unit: mm). GFRP, Glass Fiber Reinforced Polymer.

**Figure 9:**
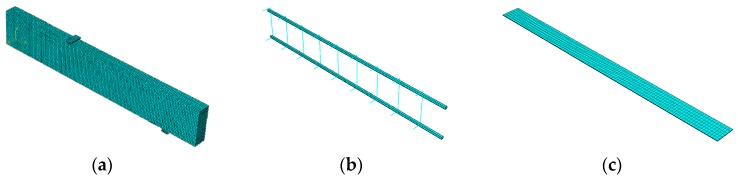
FE model: (**a**) concrete; (**b**) steel; (**c**) FRP.

The experiment this paper simulated was carried out by Ou [17]. The size of the reinforced concrete beam (Figure 8) was 150 mm × 250 mm × 2800 mm. In the test of Ou [17], the bending performance of the reference beam and C/GFRP strengthened RC beam with a prestress level of 30% was compared. Concrete longitudinal bars and HFRP were modeled by eight-node element C3D8R (An 8-node linear brick, reduced integration, hourglass control). The stirrups were modeled by truss element T3D2 (A 2-node linear 3-D truss). C/GFRP and RC beams were bonded by cohesive element COH3D8 (An 8-node three-dimensional cohesive element). The mechanical properties of component materials are shown in Table 1, Table 2, Table 3, Table 4 and Table 5. Banjara et al. [25] reported for an FRP strengthened beam that there is no size effect when the mesh size is below 25 mm. Therefore, a mesh size of 10 mm was chosen for the whole model, as shown in Figure 9.

The whole simulation process was the same as the test of Ou [17]. The beam was pre-cracked first and then strengthened with prestressed C/GFRP and subjected to static loading until failure. The Newton–Raphson iteration algorithm was used. Prestress of C/GFRP was simulated by thermal stress. Since the prestress of FRP was applied only in the longitudinal direction, the expansion coefficient was set to be a minimum value in other directions. The hybrid ratio and prestress level of C/GFRP were introduced as the influencing factors. The commonly used prestress level is about 20–50%. The hybrid ratio was determined according to reference [23]. The modelling scheme is shown in Table 6. 

## 4. Results and Discussion

### 4.1. Pre-Cracking and Strengthening Analysis

**Figure 10:**
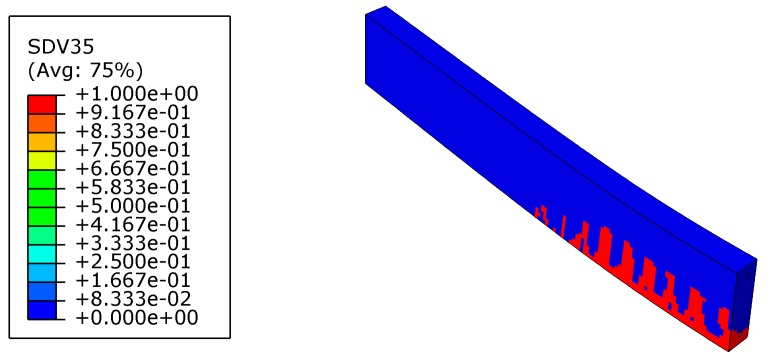
Crack distribution of the beam after pre-cracking (SDV35 = 1 stands for damage).

**Figure 11:**
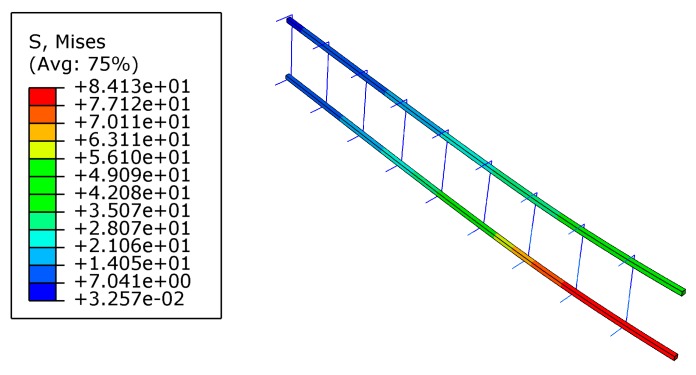
Von Mises stress of steel after pre-cracking (unit: MPa).

In order to simulate the damaged bridge that needs strengthening, the beam was first loaded to crack. The pre-cracking load was 24 kN (about 50% of the ultimate load of the reference beam). Crack distribution and the von Mises stress of steel after pre-cracking are shown in Figure 10 and Figure 11, respectively. The maximum crack length was about 60% of the beam height. The maximum Mises stress of steel in the tension zone was about 30% of its yield strength.

**Figure 12:**
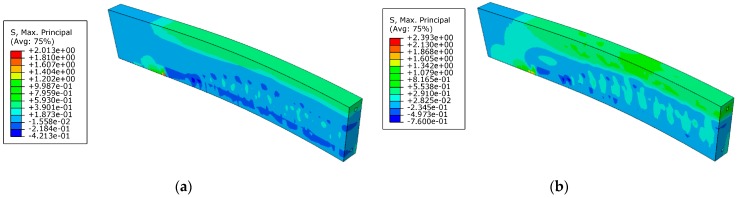
Maximum principal stress of concrete after being strengthened with prestressed CG11: (**a**) prestress level 50%; (**b**) prestress level 60% (unit: MPa).

After pre-cracking, the beam was strengthened by FRP. It is noted that higher prestress level led to greater stress concentration at the end of FRP, as shown in Figure 12. For example, when the beam was strengthened by CG11 with a prestress level of 60%, the tensile stress of concrete at the FRP end exceeded its tensile strength and cracking occurred. Therefore, the prestress of CG11 should not exceed 60%.

### 4.2. Flexural Bearing Capacity and Deflection of the Beam

**Figure 13:**
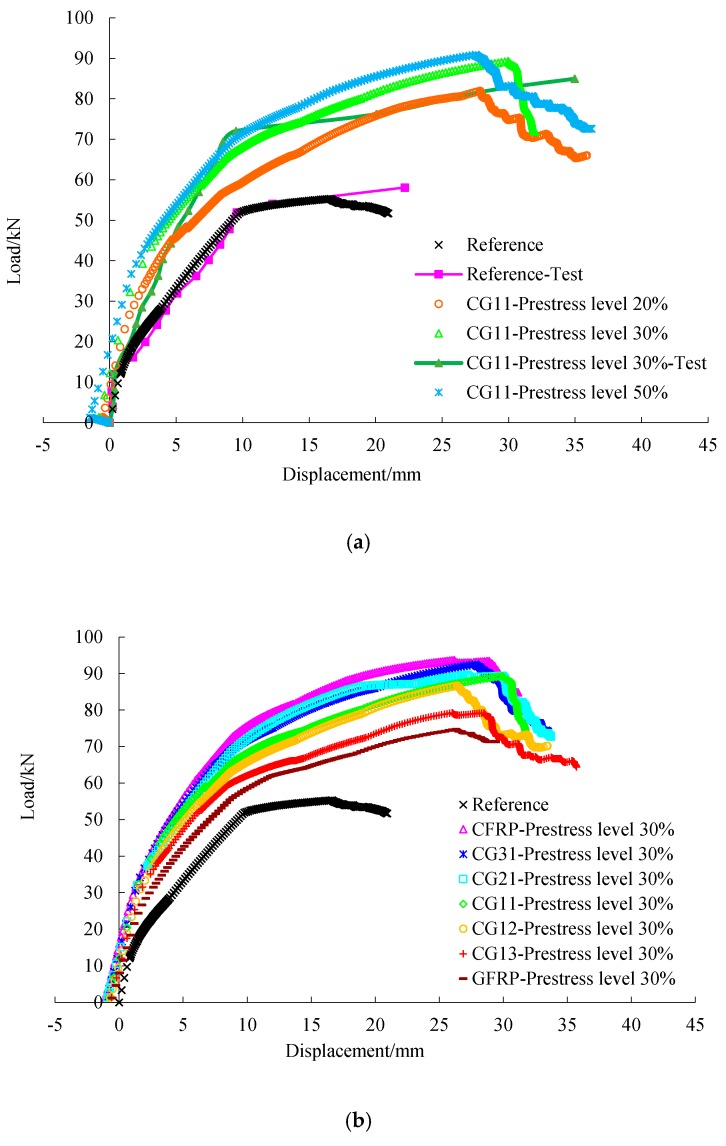
Load-deflection curves: (**a**) comparison between different prestress levels; (**b**) comparison between different hybrid ratios.

The load deflection curves and flexural capacity of the beam obtained from the test and numerical simulation are compared in Figure 13. Negative displacement obtained from the numerical simulation was caused by prestress of FRP. There was good agreement between the numerical simulation results and test data for both the reference beam and that strengthened in the slope and knee point. For example, the yield load and ultimate load in the numerical simulation were 1.04- and 0.84-times those of the test beam for the reference beam, 0.84- and 1.06-times for the beam strengthened by CG11 with a prestress level of 30%. It can be seen from Figure 13 and Table 7 that both the yield load and the ultimate load of the strengthened beams were improved significantly after being strengthened by prestressed C/GFRP. The increase of yield load helps to improve the performance of beams in normal service state. For CG11, the yield load and ultimate load of strengthened beams increased greatly when the prestress level increased from 20% to 30%, but the increase rate of yield load and ultimate load slowed down when the prestress level increased from 30% to 50%. In addition, if the prestress level of C/GFRP was too large, small deformation of the beam would lead to premature fracture of C/GFRP and a decrease of the bearing capacity of the beam. Therefore, the prestress level of C/GFRP should not be too high. At the same prestress level, the higher the carbon fiber content in C/GFRP and the higher the yield load and the ultimate load, the less the ductility, where ductility is defined as the ratio of ultimate deflection to yield deflection.

### 4.3. Stress Analysis

**Figure 14:**
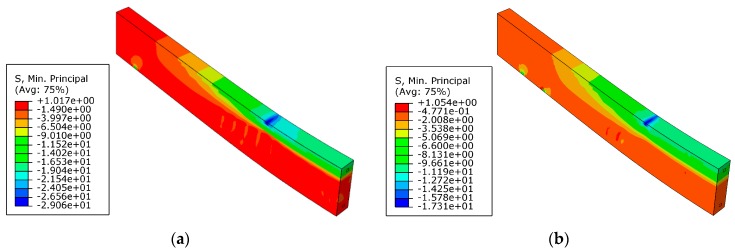
Minimum principal stress of concrete for the reference beam and strengthened beam under a load of 50 kN: (**a**) reference beam; (**b**) CG11 strengthened beam with prestress level 33% (unit: MPa).

**Figure 15:**
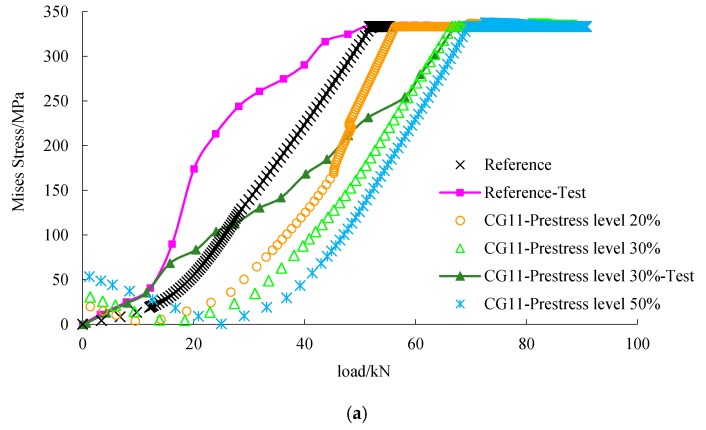

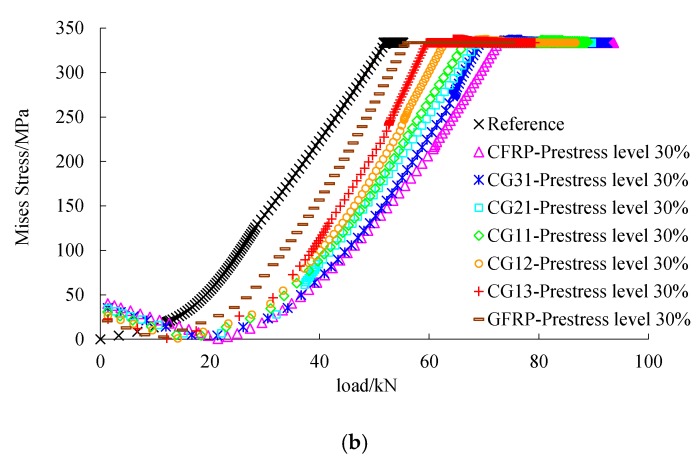
Load-Mises stress curves of the steel bar in the tensile zone: (**a**) comparison between different prestress levels; (**b**) comparison between different hybrid ratios.

**Figure 16:**
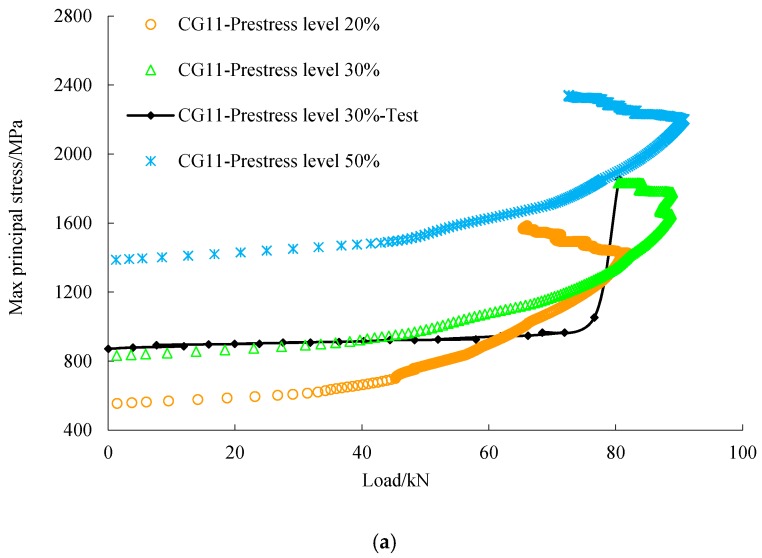

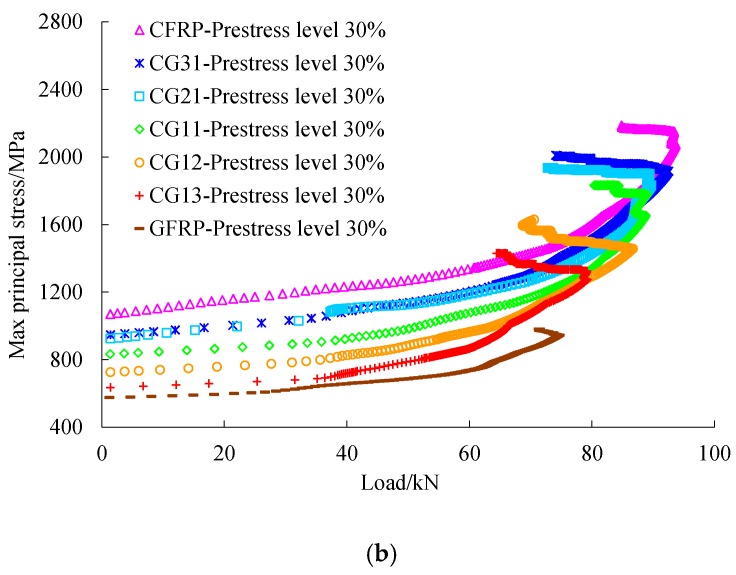
Load-max principal stress of FRP: (**a**) comparison between different prestress levels; (**b**) comparison between different hybrid ratios.

As shown in Figure 14, under the same load, the stress of the concrete of the strengthened beam was less than that of the reference beam. This indicates that prestressed FRP helps to unload the concrete. It can be seen from Figure 13, Figure 15 and Figure 16 that the load-steel stress curves, load-FRP stress curves, and load-deflection curves can be divided into three stages. In the initial stage, the deflection of the beam was small and the strain of FRP low, so the influence of FRP on the bearing capacity of the beam was limited. The load-deflection curve of the reference beam and the strengthened beam was similar, showing a linear relationship.

In the second stage, with the increase of load, the concrete in the tension zone withdrew from work because of cracking, and the tension stress was undertaken by the steel bar and C/GFRP; the stiffness of the beam obviously decreased. With the development of cracks, the rate of deflection was faster, which led to the change of the slope of the load-deflection curve. As shown in Figure 15, steel stress of the strengthened beam under the same load was smaller than that of the reference beam. This indicates that C/GFRP played an important part in unloading of the steel bar in this stage.

In the third stage, the stress of C/GFRP increased greatly after the steel bar yielded, and the slope of the load-deflection curve changed obviously. C/GFRP played a major role in restraining the beam deformation; therefore, the deflection of the strengthened beams under the same load was less than that of the reference beam. Under the same prestress level, the higher the carbon content in C/GFRP, the smaller the deflection, which indicated that the stiffness of the strengthened beam was significantly improved. However, considering the ductility and the residual strength of FRP when the beam failed, the most appropriate one was CG11. The stress-load curve of steel and C/GFRP obtained by numerical simulation was similar to the development trend of the test curve [17], and the numerical value was relatively close to the test value.

### 4.4. Failure Mode Analysis

**Figure 17:**
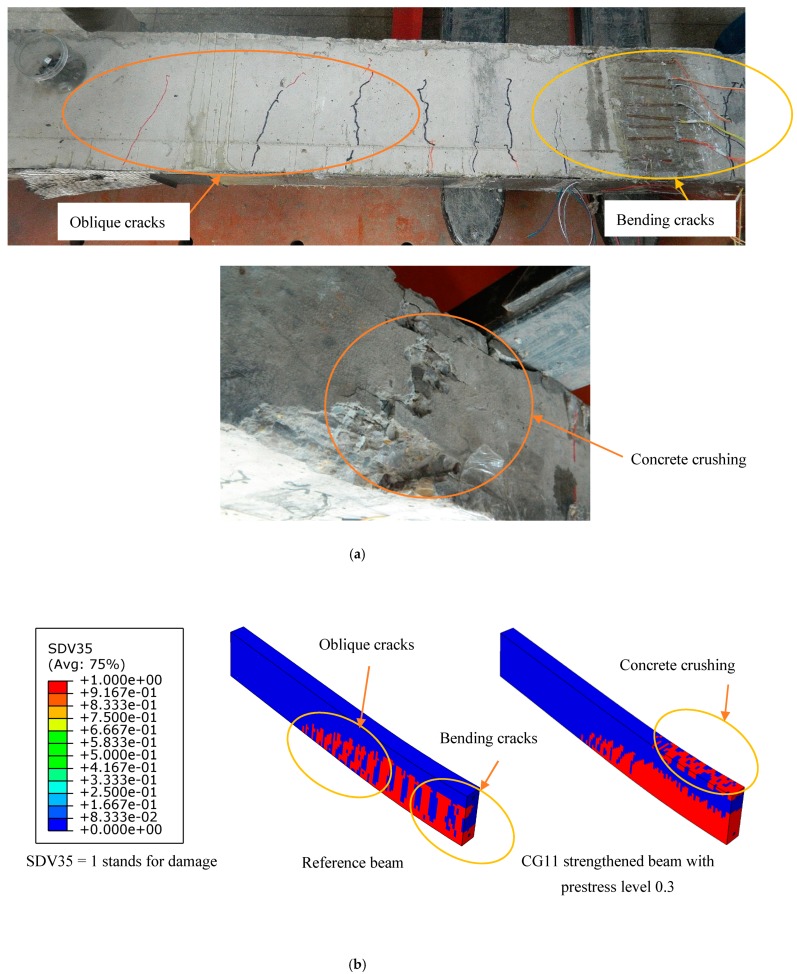
Crack distribution of the concrete of the reference beam and strengthened beam under ultimate load: (**a**) test results; (**b**) numerical simulation results.

**Figure 18:**
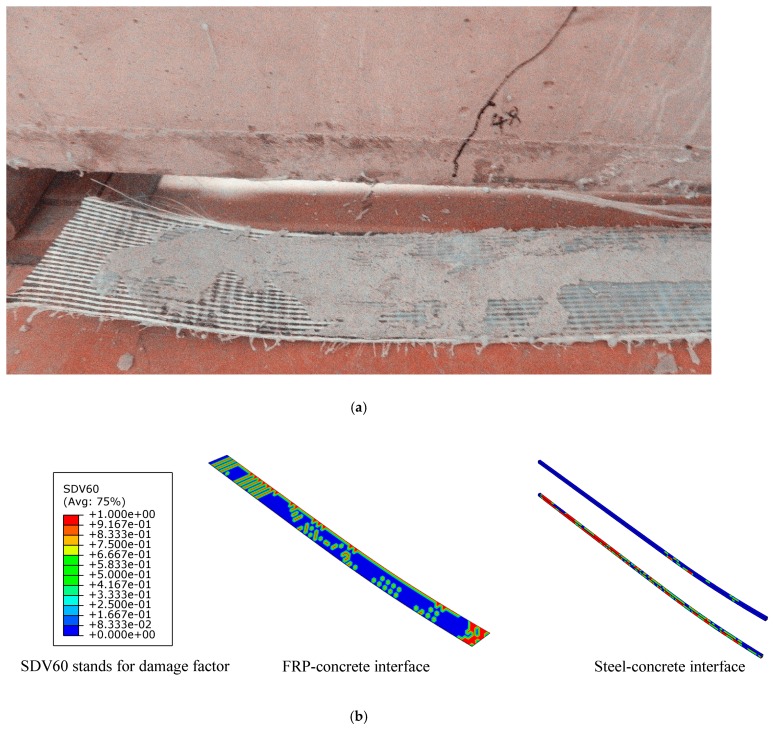
Debonding of the interface under the ultimate load of the beam strengthened with CG11 at prestress level 0.3: (**a**) debonding of the FRP-concrete interface in the test; (**b**) debonding of the interface in numerical simulation.

The failure mode observed in the test and numerical simulation is shown in Figure 17 and Figure 18. The numerical model could simulate all failure modes of the test beams. The failure mode of reference beams was steel bar yielding. The failure mode of prestressed C/GFRP strengthened beams was concrete crushing in the compression zone and FRP peeling failure [17]. Figure 17 shows that the crack patterns obtained in the test and numerical simulation were similar, which indicated that the model proposed in this paper could capture the mechanisms of fracture in the beams. The cohesive model proposed in this paper could simulate debonding, which the experiments showed, as illustrated in Figure 18. Debonding of C/GFRP mainly took place in the area with large bond slip caused by bending cracks. Damage of the steel-concrete interface was caused by concrete crack and crush, which led to the slide of the bond.

## 5. Conclusions

In this paper, the bending behavior of RC beams strengthened with prestressed hybrid FRP was studied. The elastic-plastic damage constitutive model of concrete and the mixed mode cohesive model of the interface were introduced into the numerical simulation by the UMAT subroutine provided by Abaqus. The results of the numerical simulation agreed well with the experimental results such as load-deflection curve, ultimate flexural capacity, ductility, and failure modes. The hybrid ratio and prestress level of C/GFRP were introduced as the influencing factors. The yield load, ultimate load, stress development of C/GFRP and steel bar, and deflection development were analyzed. The strengthening effect was evaluated by the improvement of bending capacity, stiffness, and ductility. The main conclusions are as follows:

(1) Compared with the reference beam, the bearing capacity of beams strengthened by prestressed C/GFRP was significantly improved, and the method of pre-stressed C/GFRP strengthening was an effective active strengthening method for damaged bridges.

(2) For the beam simulated in this paper, the characteristic loads of the prestressed GFRP strengthened beam were the lowest ones. The yield load of the prestressed C/GFRP strengthened beam increased with carbon fiber content in C/GFRP. However, the carbon fiber content had no significant effect on the ultimate load of the beam. Compared with CG11, the residual strength of CG31 and CG21 was large, which was unnecessarily excessive for high strength materials, and the ductility of the strengthened beam was relatively low. After comparison of the capacity and ductility of the beam strengthened by C/GFRP with different carbon content, CG11 was the best mixing ratio. 

(3) For CG11 strengthened beams in this paper, it was appropriate to keep the prestress level between 30 and 50% of the ultimate strength. It should be noted that a higher prestress level leads to greater stress concentration at the end of C/GFRP, which may cause stress concentration and cracks. In addition, if the prestress level of C/GFRP was too large, small deformation of the beam would lead to premature fracture of C/GFRP and decrease the bearing capacity of the beam. Therefore, the prestress level of C/GFRP should not be too high. In addition, effective anchoring methods can be considered to reduce the impact of debonding on the beam.

## Figures and Tables

**Table 1:** Material properties of concrete.

Status	Elastic Modulus (GPa)	Poisson Ratio	Yield Strength (MPa)	Ultimate Strength (MPa)	*α*	*S*	*ϕ*
Compression	31.0	0.2	30.50	33.16	0.1	5.5 × 10^−5^	1
Tension	31.0	0.2	2.0	2.4	0.1	1.0 × 10^−10^	1

**Table 2:** Material parameters of the steel/concrete interface.

Normal Stiffness *K_n_* (MPa/mm)	Shear Stiffness *K_s_* (MPa/mm)	Traction/MPa		Fracture Energy (N·mm^−1^)		η	*k*	*γ*
		$\sigma_{n}^{0}$	$\sigma_{s}^{0}$	$G_{n}^{0}$	$G_{s}^{0}$			
14.52	11.23	3.49	8.13	2.36	45.81	1.45	1	6.85 × 10^−2^

**Table 3:** Material parameters of FRP/concrete interface.

Normal Stiffness *K_n_* (GPa/mm)	Shear Stiffness *K_s_* (GPa/mm)	Traction (MPa)		Fracture Energy (N·mm^−1^)		η	*k*	*γ*
		$\sigma_{n}^{0}$	$\sigma_{s}^{0}$	$G_{n}^{0}$	$G_{s}^{0}$			
10.00	3.85	2.71	3.03	0.07	0.28	2.10	1	6.85 × 10^−2^

**Table 4:** Material parameters of steel.

Material	Elastic Module (GPa)	Poisson Ratio	Yield Strength (MPa)
Longitudinal steel bar	200.0	0.3	333.5
Stirrup	210.0	0.3	207.7

**Table 5:** Material parameters of HFRP.

Material	C	G	CG13	CG12	CG11	CG21	CG31
Elastic module/GPa	234.2	75.0	90.0	124.1	176.6	190.9	207.2
Tensile strength/MPa	3848.2	2003.2	2161.1	2349.7	2838.8	3063.5	3266.3

Note: G denotes Glass fiber reinforced polymer; CG13, CG12, CG13, CG21, CG31 denote Carbon-Glass hybrid fiber reinforced polymer with volume ratios of 1:3, 1:2, 1:1, 2:1, 3:1.

**Table 6:** Modelling scheme.

Strengthening Material	C	G	CG12	CG12	CG11	CG11	CG11	CG21	CG31
**Prestress level**	30%	30%	30%	30%	20%	30%	50%	30%	30%

Note: G denotes Glass fiber reinforced polymer, CG13, CG12, CG13, CG21, CG31 denote Carbon-Glass hybrid fiber reinforced polymer with volume ratios of 1:3, 1:2, 1:1, 2:1, 3:1.

**Table 7:** Flexural capacity of the beam.

Strengthening Material	Prestress Level	Carbon Fiber Ratio (%)	Ductility	Improvement (%)	Yield Load (kN)	Improvement (%)	Ultimate Load (kN)	Improvement (%)
None	—	—	1.62	—	52.44	—	55.27	—
C	30%	100.0	3.16	95.1	72.67	38.6	93.41	69.0
CG31	30%	75.0	2.62	61.6	69.33	32.2	89.43	61.8
CG21	30%	66.7	2.36	45.1	69.03	31.6	86.96	57.3
CG11	20%	50.0	3.33	104.9	56.34	7.4	81.93	48.2
CG11	30%	50.0	3.38	108.4	65.73	25.3	89.26	61.5
CG11	50%	50.0	3.01	85.3	69.46	32.4	90.85	64.4
CG12	30%	33.3	3.02	86.2	63.01	20.1	86.65	56.8
CG13	30%	25.0	2.76	69.9	59.96	14.3	77.99	41.1
G	30%	0.0	2.97	82.7	56.03	6.8	74.70	35.2

## References

[1] Attari N., Amziane S., Chemrouk M. (2012). Flexural strengthening of concrete beams using CFRP, GFRP and hybrid FRP sheets. Constr. Build. Mater..

[2] Hawileh R.A., Rasheed H.A., Abdalla J.A., Al-Tamimi A.K. (2014). Behavior of reinforced concrete beams strengthened with externally bonded hybrid fiber reinforced polymer systems. Mater. Des..

[3] Xiong G.J., Yang J.Z., Ji Z.B. (2004). Behavior of Reinforced Concrete Beams Strengthened with Externally Bonded Hybrid Carbon Fiber–Glass Fiber Sheets. J. Compos. Constr..

[4] Chen X., Li H., Yang Y. (2012). Experimental study on flexural fatigue performance of RC beams stengthened with HFRP. Ind. Constr..

[5] He X., Guo X., Li Y., Shen Q. (2014). Strengthening mechanism of concrete beam strengthened with externally bonded interply hybrid GFRP/CFRP. J. Huazhong Univ. Sci. Technol..

[6] Hu H.-T., Lin F.-M., Jan Y.-Y. (2004). Nonlinear finite element analysis of reinforced concrete beams strengthened by fiber-reinforced plastics. Compos. Struct..

[7] Zhang A.H., Jin W.L., Li G.B. (2006). Behavior of preloaded RC beams strengthened with CFRP laminates. J. Zhejiang Univ..

[8] Bennegadi M., Sereir Z., Amziane S. (2013). 3D nonlinear finite element model for the volume optimization of a RC beam externally reinforced with a HFRP plate. Constr. Build. Mater..

[9] Hawileh R.A. (2012). Nonlinear finite element modeling of RC beams strengthened with NSM FRP rods. Constr. Build. Mater..

[10] Xu L., Chi Y., Su J., Xia D. (2008). Nonlinear finite element analysis of steel fiber reinforced concrete deep beams. Wuhan Univ. J. Nat. Sci..

[11] Obaidat Y.T., Heyden S., Dahlblom O. (2010). The effect of CFRP and CFRP/concrete interface models when modelling retrofitted RC beams with FEM. Compos. Struct..

[12] Contrafatto L., Cuomo M. (2006). A framework of elastic–plastic damaging model for concrete under multiaxial stress states. Int. J. Plast..

[13] Sun W., Huang Y. (2016). Anisotropic Nonlinear Elastic Model of Concrete and Secondary Development in ABAQUS. Open Civ. Eng. J..

[14] Lemaitre J., Desmorat R., Sauzay M. (2000). Anisotropic damage law of evolution. Eur. J. Mech. -A/Solids.

[15] Shen X.P., Wang X.C. (2014). A plastic damage model with stress triaxiality-dependent hardening for concrete. Comput. Mater. Contin..

[16] Shen X., Shen G., Zhou L. (2006). A stress-triaxiality-dependent plastic damage model for concrete. Eng. Mech..

[17] Ou S. (2015). Study on Flexural Performance of RC Beams Strengthened with Prestressed C/GFRP Sheets.

[18] Park K., Paulino G.H. (2011). Cohesive Zone Models: A Critical Review of Traction-Separation Relationships Across Fracture Surfaces. Appl. Mech. Rev..

[19] Belnoue J.P.-H., Giannis S., Dawson M., Hallett S.R. (2016). Cohesive/adhesive failure interaction in ductile adhesive joints Part II: Quasi-static and fatigue analysis of double lap-joint specimens subjected to through-thickness compressive loading. Int. J. Adhes. Adhes..

[20] De Lorenzis L., Fernando D., Teng J.-G. (2013). Coupled mixed-mode cohesive zone modeling of interfacial debonding in simply supported plated beams. Int. J. Solids Struct..

[21] Wang X., Zhou C. (2018). Numerical investigation for the flexural strengthening of reinforced concrete beams with external prestressed HFRP sheets. Constr. Build. Mater..

[22] Qin R., Zhou A., Lau D. (2017). Effect of reinforcement ratio on the flexural performance of hybrid FRP reinforced concrete beams. Compos. Part B: Eng..

[23] Lianyungang Highway Administration Office, Nanjing University of Aeronautics and Astronautics, Nanjing Kening Civil Engineering Co., Ltd. (2013). Research on Key Technologies of Prestressed Hybrid CFRP/GFRP Sheet in Strengthening of Concrete Bridges.

[24] Shadan F., Khaloo A., Shadan P. (2015). Numerical study on flexural strengthening of squat RC shear wall using FRP laminates. Sci. Iran..

[25] Banjara N.K., Ramanjaneyulu K. (2017). Experimental and numerical investigations on the performance evaluation of shear deficient and GFRP strengthened reinforced concrete beams. Constr. Build. Mater..

